# Assessment of right–left differences in trabecular bone structure within the maxillary lateral incisor–canine region using fractal analysis

**DOI:** 10.1186/s12903-026-09728-y

**Published:** 2026-08-26

**Authors:** Hilal Algul, Gozde Haksayar, Tulin Ufuk Toygar Memikoglu, Ayse Tuba Altug

**Affiliations:** 1 Department of Orthodontics, Ankara University Faculty of Dentistry, Incitas Street, Besevler, Yenimahalle, Ankara, 06560 Turkey; 2 Department of Oral, Dental and Maxillofacial Radiology, Ankara University Faculty of Dentistry, Ankara, Turkey

**Keywords:** Bone microstructure, Fractal analysis, Maxilla, Panoramic radiography, Right–left asymmetry, Trabecular bone

## Abstract

**Background:**

Maxillary lateral incisor agenesis, peg-shaped lateral incisors, impacted canines, and cleft lip and palate frequently involve the maxillary lateral incisor–canine region and often show side-related distribution patterns. Whether trabecular bone structure within this region differs between sides in clinically healthy individuals remains unclear. This study evaluated right–left differences in maxillary trabecular bone structure using fractal analysis.

**Methods:**

Panoramic radiographs of 88 adults (43 males, 45 females; 18–40 years) with Class I occlusion and no dental, skeletal, or orofacial anomalies were retrospectively analyzed. Bilateral polygon-shaped ROIs were defined between the maxillary lateral incisor and canine roots. As methodological validation, standardized 20 × 20 pixel square ROIs were additionally analyzed. FD values were calculated using the ImageJ box-counting method. Inter-observer reliability was assessed by two independent oral and maxillofacial radiologists using the intraclass correlation coefficient (ICC) and Dahlberg error. Right–left comparisons were performed using paired-samples *t*-tests or Wilcoxon signed-rank tests. Associations with age and sex were also evaluated.

**Results:**

Using anatomically defined freehand ROIs, FD values were significantly higher in the left maxillary region than in the right (0.77 ± 0.23 vs. 0.70 ± 0.25; mean paired difference = 0.07, 95% CI: 0.01–0.13; *p* = 0.024; Cohen’s *d*_z_​* = 0.24*). The same direction of the right–left difference was confirmed using standardized square ROIs. No significant right–left differences were found for ROI area (*p* = 0.229), mean gray value (*p* = 0.618), minimum gray value (*p* = 0.682), or maximum gray value (*p* = 0.708). The right–left FD difference was not associated with age or sex.

**Conclusions:**

A small but statistically significant right–left difference in FD values was detected within the maxillary lateral incisor–canine region. Although the effect size was small and the biological significance remains uncertain, consistent findings across two ROI approaches together with excellent inter-observer reproducibility support the methodological consistency of the proposed protocol. The findings should be interpreted as a subtle radiographic observation rather than as definitive evidence of physiological maxillary asymmetry.

**Clinical trial registration:**

Not applicable.

## Background

Trabecular bone microstructure is a fundamental determinant of bone strength, mechanical adaptation, and remodeling. In the craniofacial region, trabecular bone organization is shaped by the complex interplay of genetic background, growth and developmental processes, and adaptive responses to functional stimuli. The maxilla, in particular, may exhibit marked regional variation in trabecular architecture because of its unique anatomical configuration and the functional influences to which it is subjected during mastication [1, 2].

During craniofacial development, several dental and alveolar anomalies have been reported to exhibit right–left asymmetry in their distribution within the maxilla. The maxillary lateral incisor–canine region was specifically selected for the present study because it represents the anatomical area where developmental and dentoalveolar anomalies with reported side predominance, including lateral incisor agenesis, peg-shaped lateral incisors, impacted canines, and cleft lip and palate, are most frequently encountered [3–7]. Previous authors have suggested that such asymmetric distributions may reflect differences in morphogenesis between the right and left sides of the craniofacial complex during embryological development [8]. However, no direct evidence currently demonstrates that these developmental phenomena are associated with physiological differences in trabecular bone organization in clinically healthy individuals. Consequently, the present study should be regarded as an exploratory investigation of potential side-related variation rather than an attempt to establish a direct developmental relationship between anomaly distribution and trabecular architecture.

From a biological perspective, subtle side-related differences in trabecular organization could theoretically arise through multiple mechanisms. Developmental asymmetry during craniofacial morphogenesis, differences in functional loading, masticatory dominance, local vascularization, and region-specific remodeling activity may all contribute to variation in trabecular complexity. Although the potential influence of these factors on maxillary trabecular architecture remains largely unexplored, they provide a biologically plausible framework for investigating side-related variation in clinically healthy individuals.

Fractal analysis is a mathematical approach used for the quantitative assessment of trabecular bone structure through the fractal dimension (FD), which reflects the geometric complexity and organizational characteristics of trabecular bone. When applied to dental and maxillofacial radiographs as well as cone-beam computed tomography (CBCT) images, fractal analysis enables objective and reproducible quantification of subtle structural changes within trabecular bone [9]. Based on the box-counting algorithm, this method evaluates the structural organization of the trabecular network rather than relying solely on conventional density-based measurements, thereby providing a useful tool for assessing regional variation in bone structure and potential side-related differences [10].

In dental radiology, fractal analysis has been applied to panoramic, periapical, bitewing, cephalometric, and CBCT images for the assessment of osteoporosis-related changes, peri-implant bone remodeling, periodontal alterations, temporomandibular joint-related bony changes, and other local or systemic conditions affecting bone quality. A systematic review by Kato et al. reported that panoramic and periapical radiographs are among the most frequently used imaging modalities in dental fractal analysis, whereas CBCT-based applications remain comparatively fewer and methodologically more heterogeneous. More recent reviews have likewise emphasized the growing use of fractal analysis in dentomaxillofacial imaging while highlighting the influence of region-of-interest (ROI) selection, image-processing procedures, and software-related variability on the reproducibility of FD measurements [11, 12].

Compared with the extensive body of mandibular literature, studies specifically focused on the maxilla remain relatively limited. Existing investigations have mainly addressed trabecular characteristics at implant-related sites, mini-implant regions, and grafted or cleft-affected maxillary segments rather than possible right–left variation in otherwise healthy individuals. Studies using CBCT and micro-computed tomography have demonstrated that maxillary trabecular structure can be quantitatively assessed and may vary according to local anatomical and clinical conditions, supporting the biological plausibility of regional microstructural variation within the maxilla [10, 13, 14]. To the best of our knowledge, right–left differences in trabecular bone structure within the maxillary lateral incisor–canine region have not been directly investigated in clinically healthy adults using panoramic radiographs. Consequently, the normative pattern of side-related trabecular variation within this region remains insufficiently characterized.

Although CBCT provides three-dimensional information and enables more detailed evaluation of trabecular microstructure, its use in orthodontic imaging should be justified on an individual basis and should not be performed routinely in the absence of a specific diagnostic indication [15, 16]. In clinically healthy individuals without a direct need for three-dimensional imaging, panoramic radiographs represent a more ethical and clinically accessible source of data. In addition to being routinely available in orthodontic records and involving lower radiation exposure, panoramic images permit simultaneous bilateral assessment using a standardized retrospective image. Nevertheless, panoramic radiography is a two-dimensional projection technique, and FD values derived from panoramic images should not be considered directly interchangeable with CBCT-based measurements [11, 16, 17].

Therefore, the aim of this study was to evaluate right–left differences in trabecular bone structure within the maxillary lateral incisor–canine region using fractal analysis and to determine whether subtle radiographic differences could be detected in a clinically healthy adult population.

## Methods

### Study design and ethical approval

This retrospective single-center radiographic study was conducted using archived panoramic radiographs obtained from the Department of Orthodontics, Faculty of Dentistry, Ankara University, Ankara, Turkey (approval no. 14/14; October 6, 2025). The study material consisted of archived panoramic radiographs obtained from adult individuals who presented to the Department of Orthodontics, Faculty of Dentistry, Ankara University, for orthodontic evaluation. The radiographs had been acquired as part of routine initial orthodontic records after clinical examination and informed consent for diagnostic imaging. Ethical approval was obtained for the use of these archived images for research purposes. The study was conducted in accordance with the principles of the Declaration of Helsinki.

### Power analysis

An α priori power analysis was performed for the primary paired comparison of right and left maxillary fractal dimension (FD) values. As no previous studies evaluating right–left fractal dimension differences in the maxillary lateral incisor–canine region were available, a two-tailed paired-samples comparison was assumed with a significance level of α = 0.05, a target statistical power of 90%, and a small-to-moderate effect size (Cohen’s *d*_z_ = 0.35) was selected based on conventional recommendations for exploratory biological research and paired-sample study designs. Based on these parameters, the minimum required sample size was calculated as 88 individuals, corresponding to 88 paired right–left observations.

### Participants

The study sample consisted of 88 adult individuals (43 males and 45 females) with Class I occlusion and no dental, skeletal, or orofacial anomalies who presented for orthodontic evaluation. These individuals were selected from an initial archive of panoramic radiographs obtained from 136 subjects. After screening the archived radiographs according to the predefined eligibility and image-quality criteria, 48 individuals were excluded and 88 were included in the final analysis. Only radiographs with sufficient diagnostic quality and clear bilateral visualization of the maxillary alveolar region between the lateral incisor and canine roots were considered eligible for reliable ROI delineation and fractal analysis. Because bilateral ROIs were evaluated in each subject, a total of 176 ROIs were analyzed.

## Inclusion criteria

The inclusion criteria were as follows:

- age 18 years or older;
- absence of dental, skeletal, or orofacial anomalies, such as impacted teeth, missing teeth, crown–root anomalies and cleft lip and/or palate;
- relatively parallel orientation of the maxillary lateral incisor and canine roots, without marked convergence or divergence;
- no previous history of orthodontic treatment;
- no history of surgical intervention or trauma affecting the relevant maxillary regions;
- panoramic radiographs with sufficient diagnostic quality, contrast, and clear bilateral visualization of the lateral incisor–canine region to permit reliable ROI delineation and fractal analysis.

### Exclusion criteria

The exclusion criteria were as follows:

- age below 18 years;
- presence of dental, skeletal or orofacial anomalies affecting the maxilla, including impacted teeth, congenitally missing teeth, supernumerary teeth, crown–root anomalies and cleft lip and/or palate;
- moderate to severe periodontal disease or significant alveolar bone loss in the regions of interest;
- history of cystic lesions, tumors, trauma or other pathologies involving the maxilla;
- previous surgical interventions affecting the relevant maxillary regions, such as implant placement, bone grafting or apical resection;
- presence of systemic diseases known to affect bone metabolism, including osteoporosis, osteopenia and Paget’s disease;
- long-term use of corticosteroids or other medications known to affect bone metabolism;
- previous orthodontic treatment;
- presence of prosthetic appliances or fixed restorations in the regions of interest;
- panoramic radiographs of insufficient diagnostic quality or with artifacts, distortion, or inadequate bilateral visualization that could compromise reliable ROI standardization and fractal analysis.

### Procedure

As the study population consisted of clinically healthy individuals, CBCT was not considered ethically justified in the absence of a diagnostic indication in accordance with radiation protection principles; therefore, archived panoramic radiographs were used for analysis. The panoramic radiographs used in this study had been acquired using a Carestream Dental CS 8100 unit (Carestream Dental LLC, Atlanta, GA, USA).

**Fig. 1:**
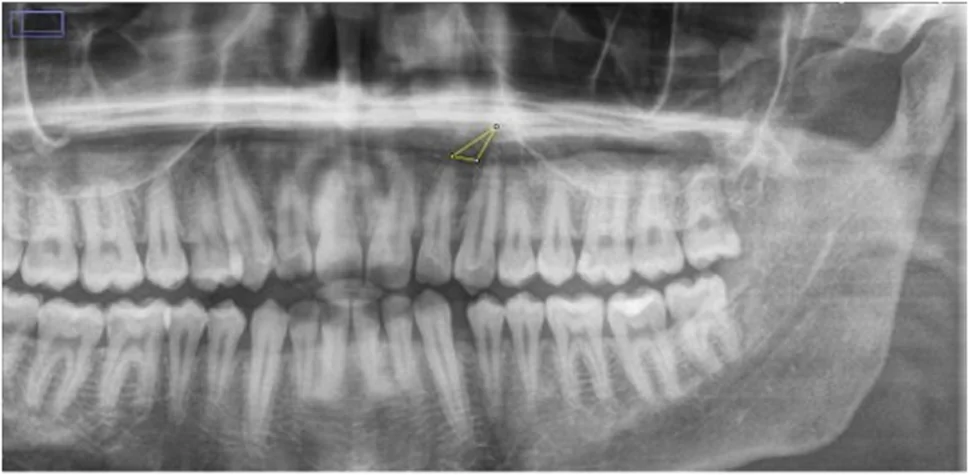
Selection of the region of interest (ROI) in the maxillary alveolar bone at the level of the lateral incisor–canine apices on the panoramic radiograph

For the primary analysis, fractal analysis was performed using ImageJ software (ImageJ 1.53e, National Institutes of Health, Bethesda, MD, USA). The selected panoramic radiographs were anonymized by removing all patient identification information and exported in TIFF format for digital analysis. All exported images had a matrix size of 1024 × 483 pixels, with a resolution of 96 dpi and a bit depth of 24 bits. For each individual, one polygon-shaped ROI was selected on each side of the maxilla, resulting in a total of 176 ROIs. ROI selection was performed within the interradicular trabecular bone between the apices of the maxillary lateral incisor and canine roots, where the anatomical boundaries were clearly identifiable, superimposition was limited, and the trabecular structure could be observed relatively homogeneously (Fig. 1). Polygonal ROIs were manually delineated to encompass the largest possible trabecular bone area while deliberately excluding adjacent tooth roots, cortical bone, the nasal cavity, the nasal floor, the anterior wall of the maxillary sinus, and other non-trabecular anatomical structures. Similar polygonal ROI approaches have previously been described in dental fractal analysis studies. To achieve bilateral standardization, the ROI drawn on one side was mirrored anatomically to the corresponding contralateral interradicular region using identical anatomical landmarks and predefined selection criteria. Because minor anatomical asymmetries may still influence the exact polygonal contour, ROI area was recorded for every selection, and right–left ROI areas were statistically compared to evaluate the consistency of ROI delineation between sides.

Recognizing that anatomically defined polygonal ROIs cannot be reproduced with completely identical geometric dimensions, an additional methodological validation was performed using standardized 20 × 20 pixel square ROIs positioned within the same bilateral interradicular region. This complementary analysis was undertaken to determine whether the direction of the observed right–left FD difference remained unchanged under a fixed geometric ROI approach and to assess the potential influence of ROI geometry on the primary findings.

**Fig. 2:**
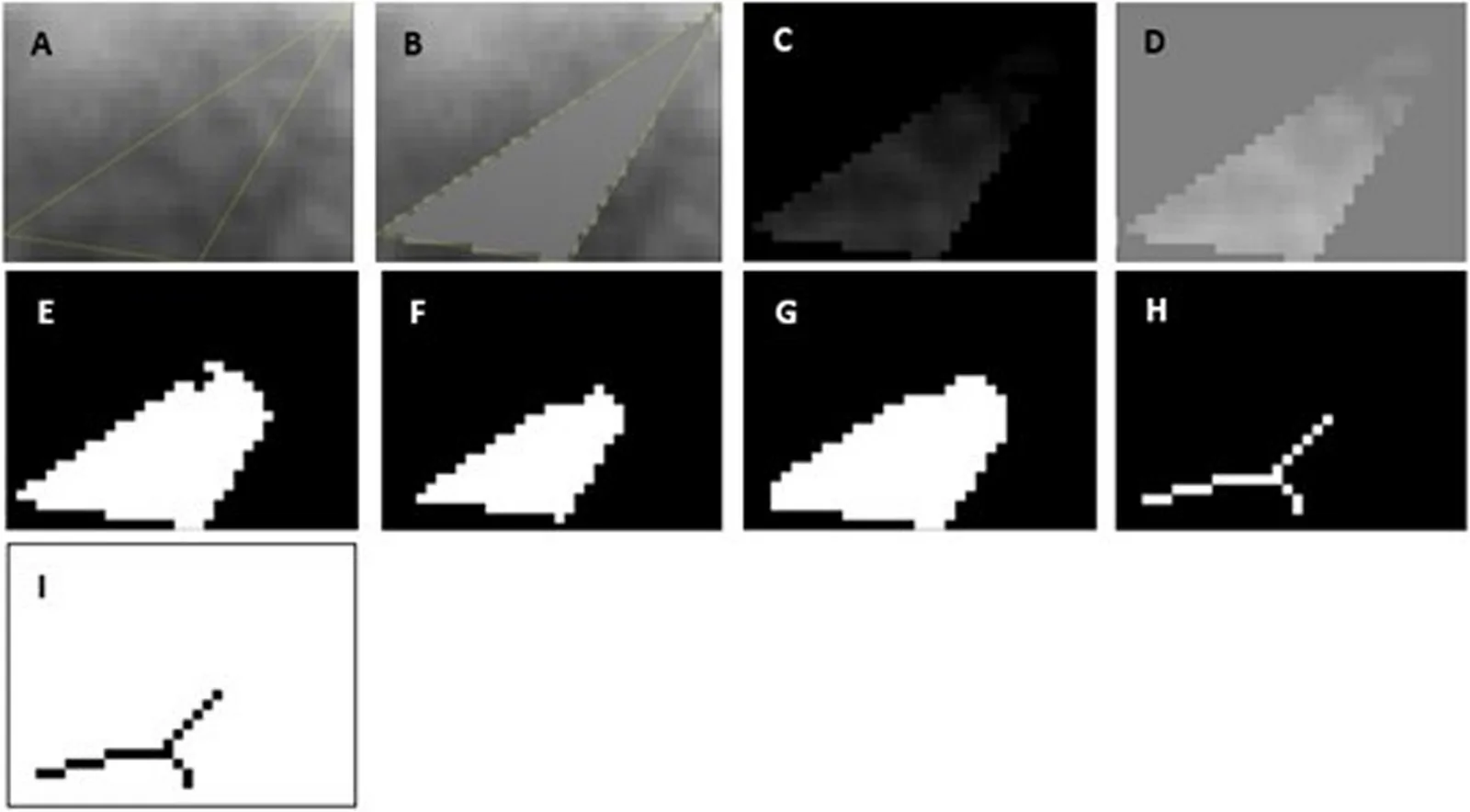
Illustration of the image preprocessing stages used for fractal dimension analysis of trabecular bone, from region-of-interest (ROI) selection to skeletonization. **A** Cropped and duplicated image. **B** Gaussian blur. **C** Subtraction of the blurred image from the duplicated image. **D **Addition of a grayscale value of 128. **E** Conversion to binary image. **F** Erosion. **G **Dilation. **H **Skeletonization. **I **Inverted image

**Fig. 3:**
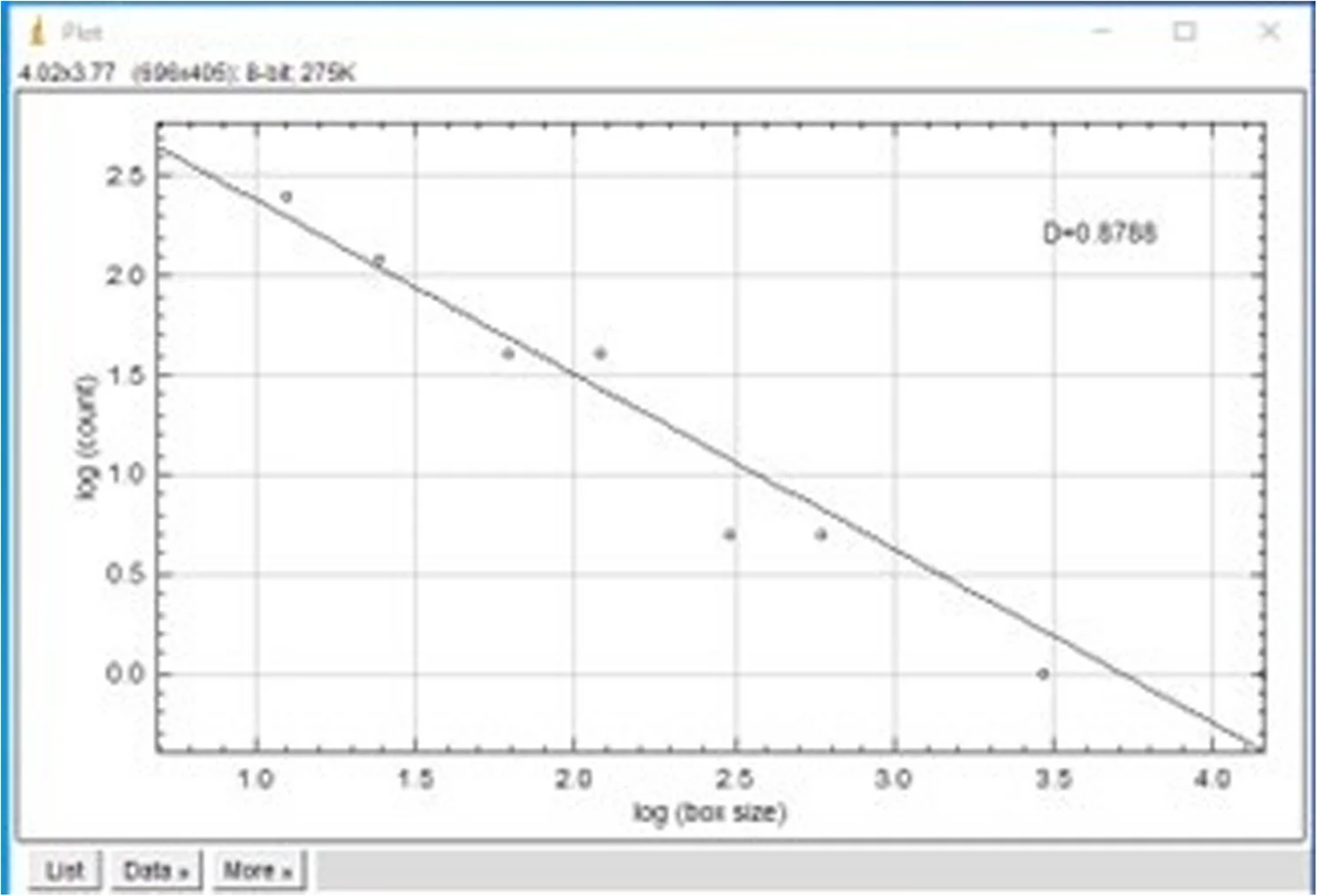
Log–log plot used to calculate the fractal dimension (FD) by the box-counting method

After ROI selection, the selected regions were cropped, duplicated, and converted to 8-bit grayscale format. A Gaussian blur filter was then applied (sigma = 35), and the blurred image was subtracted from the duplicated original image. Subsequently, a grayscale value of 128 was added, and the images were converted to binary format using a fixed threshold value of 128, in accordance with the widely used ImageJ-based fractal analysis workflow derived from the method of White and Rudolph, thereby ensuring methodological consistency with previous dental radiographic FD studies [11, 18, 19]. Morphological processing was then performed by sequential application of erosion and dilation, followed by skeletonization so that the trabecular network was represented by one-pixel-wide lines. The skeletonized image was subsequently inverted, and fractal dimension (FD) values were calculated using the standard box-counting method available in ImageJ without the use of external plugins or additional software (Figs. 2 and 3). The identical image-processing workflow was applied to both polygonal and square ROIs.

Although a fixed threshold was selected to ensure methodological consistency across all images, alternative thresholding strategies may produce different absolute FD values and therefore cannot be considered entirely interchangeable. Because the anatomical side was inherently recognizable on panoramic radiographs, blinding to side during ROI selection was not feasible. To minimize measurement bias, all ROIs were selected according to predefined anatomical criteria and processed using an identical standardized image-processing workflow.

### Measurement reliability

To assess the inter-observer reliability of the proposed fractal analysis protocol, fractal dimension (FD) measurements were independently performed by two experienced oral and maxillofacial radiologists using the same set of panoramic radiographs from 88 individuals. Each observer independently selected and delineated both the anatomically defined polygonal ROIs and the standardized 20 × 20 pixel square ROIs within the predefined bilateral interradicular region according to the established ROI selection protocol. The complete image-processing workflow, including ROI delineation, preprocessing, and FD calculation, was independently repeated by each observer.

Inter-observer agreement was evaluated using the intraclass correlation coefficient (ICC) based on a two-way random-effects, absolute-agreement, single-measurement model, with corresponding 95% confidence intervals. Measurement error was additionally assessed using Dahlberg’s formula *(ME = √(Σd²/2n))*, where *d* represents the difference between measurements obtained by the two observers and *n* is the number of paired observations.

This reliability assessment therefore reflects not only the reproducibility of fractal dimension measurements but also the consistency of independent ROI selection, delineation, and execution of the entire image-processing workflow for both ROI approaches.

### Statistical analysis

Statistical analyses were performed using SPSS Statistics software (version 22.0; IBM Corp., Armonk, NY, USA). The primary outcome of the study was the right–left difference in maxillary fractal dimension (FD) values obtained using anatomically defined polygonal ROIs. Secondary variables included ROI area, mean gray value, minimum gray value, and maximum gray value. These secondary variables were analyzed primarily as methodological control parameters supporting interpretation of FD measurements. Data distribution was assessed using the Shapiro–Wilk test. For variables showing normal distribution, right–left comparisons were performed using paired-samples *t*-tests. When the normality assumption was not met, the Wilcoxon signed-rank test was used. Associations between the right–left FD difference and age were evaluated using Pearson correlation analysis, and differences according to sex were assessed using the independent-samples *t*-test.

As an additional methodological validation, paired-samples *t*-tests were also performed to compare right and left FD values obtained using standardized 20 × 20 pixel square ROIs. Furthermore, paired comparisons between polygonal and square ROI measurements were performed separately for the right and left sides to evaluate the influence of ROI geometry on FD measurements. Inter-observer reliability was evaluated using the intraclass correlation coefficient (ICC) based on a two-way random-effects, absolute-agreement, single-measurement model. Measurement error was assessed using Dahlberg’s formula. All statistical tests were two-tailed, and the level of significance was set at α = 0.05. Results were reported as mean ± standard deviation, mean paired difference, 95% confidence interval (CI), exact *p*-values, and, where applicable, effect size. Effect size for paired comparisons was expressed as Cohen’s *d*_z_*.*

## Results

### Demographic characteristics of the participants

A total of 88 adult individuals were included in the study, comprising 43 males and 45 females. The mean age of the participants was 23.31 ± 5.28 years (range, 18–40 years). The sample showed a nearly balanced distribution according to sex.

### Measurement reliability

Inter-observer reliability was excellent for both anatomically defined freehand ROIs and standardized square ROIs (Table 1). ICC values ranged from 0.988 to 0.993 for freehand ROIs and from 0.954 to 0.966 for square ROIs. The corresponding Dahlberg errors ranged from 0.02147 to 0.02488 for freehand ROIs and from 0.03500 to 0.03900 for square ROIs. Although inter-observer agreement was slightly lower for the standardized square ROIs, both ROI approaches demonstrated excellent reproducibility, supporting the reliability of the ROI selection protocol and the subsequent fractal dimension measurements.

**Table 1:** Inter-observer agreement and method error for fractal dimension measurements obtained using freehand and square ROIs

ROI type	Region	*n*	ICC (2,1)	95% CI	Dahlberg error	Interpretation
Freehand ROI	Right	88	0.993	0.989–0.995	0.02147	Excellent
Freehand ROI	Left	88	0.988	0.983–0.993	0.02488	Excellent
Square ROI	Right	88	0.954	0.930–0.970	0.03900	Excellent
Square ROI	Left	88	0.966	0.948–0.977	0.03500	Excellent

Inter-observer reliability was evaluated using a two-way random-effects model with absolute agreement for single measurements [ICC(2,1)]. Measurement error was assessed using the Dahlberg formula: $\mathit{ME} = \sqrt{{\sum d^2}/{2n}}$

*n *number of individuals, *FD* fractal dimension, *ICC *intraclass correlation coefficient, *CI *confidence interval.

### Comparison of fractal dimension values between the right and left maxillary regions

Using anatomically defined freehand ROIs, fractal dimension (FD) values differed significantly between the two sides of the maxilla. The left maxillary region showed higher FD values than the right region (0.77 ± 0.23 vs. 0.70 ± 0.25, respectively; *p* = 0.024) (Table 2). The mean paired difference (left–right) was 0.07 (95% CI: 0.01 to 0.13), corresponding to a small effect size (Cohen’s *d*_z_ = 0.24).

**Table 2:** Paired comparison of left and right maxillary fractal dimension values and ROI-related parameters

Variable	Right mean ± SD	Left mean ± SD	Mean paired difference (Left-Right)	SD of paired difference	95% CI of difference	t	*p*	Cohen’s *d*_z_
Freehand ROI fractal dimension (FD)	0.70 ± 0.25	0.77 ± 0.23	0.07	0.29	0.01 to 0.13	2.295	0.024	0.24
Square ROI fractal dimension (FD)	0.83 ± 0.19	0.94 ± 0.19	0.11	0.21	0.07 to 0.16	5.015	< 0.001	0.53
ROI area	430.08 ± 481.11	465.74 ± 589.42	35.66	276.33	-22.85 to 94.17	1.211	0.229	0.13
Mean gray value	92.76 ± 32.43	90.90 ± 33.18	-1.85	34.67	-9.24 to 5.52	-0.501	0.618	0.05
Minimum gray value	53.54 ± 27.92	54.89 ± 33.17	1.35	30.83	-5.18 to 7.88	0.411	0.682	0.04
Maximum gray value	140.95 ± 37.60	142.40 ± 30.19	1.44	36.01	-6.21 to 9.11	0.376	0.708	0.04

Effect size for paired comparisons was expressed as Cohen’s *d*_z_. Square ROI measurements were obtained using standardized 20 × 20 pixel regions of interest. Mean paired differences were calculated as left minus right

*SD *standard deviation, *FD *fractal dimension, *CI *confidence interval, *ROI *region of interest.

*p* values were obtained using paired-samples t-tests

The additional methodological validation using standardized 20 × 20 pixel square ROIs demonstrated the same direction of the right–left difference. FD values remained significantly higher in the left maxillary region than in the right (0.94 ± 0.19 vs. 0.83 ± 0.19, respectively; *p* < 0.001), with a mean paired difference of 0.11 (95% CI: 0.07 to 0.16) and a moderate effect size (Cohen’s *d*_z_ = 0.53) (Table 2).

### ROI area and grayscale parameters of the right and left maxillary regions

No statistically significant right–left differences were observed for ROI area or grayscale parameters, whereas a significant difference was detected only for FD values (Table 2). The mean ROI area was 430.08 ± 481.11 on the right and 465.74 ± 589.42 on the left (*p* = 0.229). The mean gray value was 92.76 ± 32.43 on the right and 90.90 ± 33.18 on the left (*p* = 0.618). The minimum gray value was 53.54 ± 27.92 on the right and 54.89 ± 33.17 on the left (*p* = 0.682), while the maximum gray value was 140.95 ± 37.60 on the right and 142.40 ± 30.19 on the left (*p* = 0.708) (Table 2). No statistically significant right–left differences were detected in ROI area or grayscale parameters under the applied analytical protocol.

### Relationship between age and left-right differences

No statistically significant association was found between age and the left–right difference in fractal dimension (*r* = 0.156, *p* = 0.146). Similarly, no significant correlations were observed between age and the left–right differences in the other radiographic parameters (Table 3).

**Table 3:** Correlation analysis between age and left-right differences in maxillary fractal dimension and ROI-related radiographic parameters

Correlations analysis between age and left-right differences					
		**Left-Right Region FD**	**Left-Right ROI Area Difference**	**Left-Right Mean Gray Value Difference**	**Left-Right Minimum Gray Value Difference**
Age	r	0.156	0.158	-0.172	-0.099
	*p*	0.146	0.420	0.108	0.357
	n	88	88	88	88

Correlation coefficients were calculated using Pearson correlation analysis. Differences were calculated as left minus right

*n *number of individuals, *FD *fractal dimension, *ROI *region of interest

### Evaluation of left-right fractal dimension differences according to sex

The left-right difference in fractal dimension did not differ significantly between male and female participants (male: 0.10 ± 0.27; female: 0.04 ± 0.31; *p* = 0.366) (Table 4).

**Table 4:** Comparison of left-right differences in maxillary fractal dimension and associated radiographic parameters by sex

Variable	Male mean ± SD (*n* = 43)	Female mean ± SD (*n* = 45)	t	*p*	Cohen’s *d*_z_
Left-Right FD difference	0.10 ± 0.27	0.04 ± 0.31	0.909	0.366	0.19
Left-Right ROI area difference	58.12 ± 375.65	14.20 ± 123.78	0.743	0.459	0.16
Left-Right mean gray value difference	-2.66 ± 34.90	-1.07 ± 34.83	-0.214	0.831	0.05
Left-Right minimum gray value difference	0.79 ± 34.36	1.88 ± 27.42	-0.165	0.869	0.04
Left-Right maximum gray value difference	0.88 ± 34.15	1.98 ± 38.08	-0.142	0.888	0.03

Differences were calculated as left minus right. Comparisons between males and females were performed using the independent-samples t-test. Effect size was expressed as Cohen’s *d*_z_

*n *number of individuals, *SD *standard deviation, *FD *fractal dimension, *ROI *region of interest.

### Effect of ROI geometry on fractal dimension measurements

To further evaluate the influence of ROI geometry on fractal dimension measurements, FD values obtained using anatomically defined freehand ROIs were compared with those obtained using standardized 20 × 20 pixel square ROIs (Table 5). On both the right and left sides, square ROIs yielded significantly higher FD values than freehand ROIs (both *p* < 0.001). The mean paired differences were 0.13 (95% CI: 0.09–0.17; *Cohen’s d*_z_* =* 0.73) for the right side and 0.17 (95% CI: 0.13–0.20; *Cohen’s d*_z_* =* 1.04) for the left side.

**Table 5:** Effect of ROI geometry on fractal dimension measurements

Comparison	Freehand ROI mean ± SD	Square ROI mean ± SD	Mean paired difference (S − F)	SD of paired difference	95% CI	t	*p*	Cohen’s *d*_z_
Right region	0.70 ± 0.25	0.83 ± 0.19	0.13	0.18	0.09 to 0.17	6.881	< 0.001	0.73
Left region	0.77 ± 0.23	0.94 ± 0.19	0.17	0.16	0.13 to 0.20	9.803	< 0.001	1.04

Positive values indicate higher fractal dimension values obtained using standardized square ROIs. Mean paired differences were calculated as square ROI minus freehand ROI

*SD *standard deviation, *ROI *region of interest, *F* Freehand, *S *Square, *CI *confidence interval

## Discussion

Although numerous studies have evaluated the trabecular microstructure of craniofacial bones using fractal analysis, investigations specifically examining right–left differences in trabecular organization within the maxilla remain limited. Most previous research has focused primarily on the application of fractal analysis for bone microstructure assessment, whereas regional or bilateral comparisons have generally been reported only as secondary findings, leaving uncertainty regarding the quantitative characterization of side-related variation in maxillary trabecular organization. Furthermore, considerable methodological heterogeneity exists with respect to imaging modality, anatomical site, ROI definition, image preprocessing sequence, thresholding strategy, and software workflow, limiting direct comparison of absolute FD values across studies [11, 12, 17, 20, 21]. Therefore, the present findings should be interpreted primarily as an internal bilateral comparison obtained under a standardized protocol rather than as values that can be directly benchmarked against previously published data.

The absolute FD values observed in the present study were lower than those reported in several previous panoramic radiographic investigations. However, direct comparison across studies should be approached cautiously because FD measurements are highly sensitive to ROI definition, image-processing workflows, thresholding strategies, imaging modality, and software implementation. Consequently, differences in methodology rather than biological variation alone may account for the discrepancies in reported absolute FD values.

Although the study population consisted predominantly of young adults, the inclusion of participants older than 35 years warrants consideration of possible age-related changes in bone microstructure, particularly in women. Previous studies have shown that osteoporosis-related alterations in craniofacial trabecular pattern can be detected by radiographic fractal analysis [22]. However, no significant associations were found between fractal dimension and age or sex in the present study. Together with the exclusion of individuals with systemic conditions affecting bone metabolism, these findings suggest that the observed side-related difference is unlikely to be primarily explained by osteoporotic or hormonally mediated trabecular variation.

The principal finding of the present study was that fractal dimension values were significantly higher on the left side of the maxilla than on the right. Because an increase in fractal dimension reflects greater trabecular structural complexity and organization rather than bone density alone, this finding suggests a subtle radiographic difference in trabecular organization within the investigated region. The detection of this difference in individuals without clinically evident dental, skeletal, or orofacial anomalies indicates that such variation may be present even in clinically healthy adults. Importantly, the observed effect size was small (Cohen’s *d < sub> z</sub > =* 0.24), indicating that the magnitude of the difference was modest. Although statistically significant, its biological and clinical relevance remains uncertain. Accordingly, these findings should be interpreted as a subtle radiographic observation identified under a standardized methodological protocol rather than as definitive evidence of physiological maxillary asymmetry. Further studies are required to determine whether the observed difference reflects underlying biological variation or residual methodological variability despite the use of a standardized protocol.

Beyond studies employing fractal analysis, there is evidence suggesting that maxillary bone structure may exhibit non-homogeneous organization. Morphometric and micro-computed tomography-based investigations have shown that trabecular architecture varies according to anatomical region and that bone density and trabecular organization are not fixed parameters [23, 24]. Moreover, morphological analyses conducted in the transverse plane have demonstrated dimensional and structural asymmetries between the right and left halves of the maxilla even in individuals considered clinically symmetrical [25]. Collectively, these findings suggest that subtle side-related variation may exist at multiple structural levels of the craniofacial complex. However, the present findings do not permit definitive conclusions regarding physiological maxillary asymmetry, and their biological significance remains uncertain. Nevertheless, they are consistent with the broader concept that apparent clinical symmetry does not necessarily indicate complete symmetry at the tissue level.

Previous studies have reported that several dental and alveolar anomalies exhibit left-side predominance within the maxilla, including cleft-related anomalies, congenital tooth agenesis, impacted canines, and peg-shaped lateral incisors [3–7]. Radiographic and CBCT-based studies in individuals with such anomalies have demonstrated alterations in trabecular bone density and organization within the affected maxillary regions [8, 26, 27]. In the present study, higher fractal dimension values were also detected on the left side in clinically healthy individuals. However, these findings should not be interpreted as evidence of an etiologic, developmental, or biological relationship between FD asymmetry and anomaly formation. Because this was a cross-sectional study including only clinically healthy adults, the findings are limited to demonstrating a side-related difference in maxillary trabecular organization within this population. Whether the observed difference has developmental or biological relevance to the distribution of these anomalies remains uncertain and cannot be determined from the present data.

From an orthodontic and craniofacial perspective, the present findings suggest that subtle side-related variation may also be detectable at the trabecular level of the maxilla. However, because the study was based on cross-sectional panoramic radiographs obtained from clinically healthy adults, these findings should not be interpreted as direct evidence of developmental or biological asymmetry [28, 29].

Side-related differences in trabecular organization may have potential biomechanical relevance because trabecular microstructure has been associated with local remodeling behavior and bone response under loading [30, 31]. However, the present study was not designed to evaluate orthodontic biomechanics or treatment-related outcomes. Therefore, any clinical implications remain speculative and require confirmation in dedicated clinical studies. At present, the findings are primarily relevant to the radiographic interpretation of maxillary symmetry rather than to clinical decision-making.

The absence of statistically significant differences between the right and left maxillary regions with respect to ROI area and mean, minimum, and maximum grayscale values supports the methodological consistency of the primary freehand ROI protocol. Although square or rectangular ROIs are frequently preferred in fractal analysis studies, the anatomically complex region between the apices of the maxillary lateral incisor and canine roots is influenced by the nasal floor, the anterior wall of the maxillary sinus, dental roots, and surrounding cortical bone [18, 32]. Therefore, anatomically defined polygonal ROIs were selected to encompass the largest representative trabecular area while deliberately excluding these adjacent structures. To minimize potential bias related to ROI size, the ROI drawn on one side was anatomically replicated on the contralateral side using identical predefined selection criteria, and bilateral ROI areas were statistically compared. Although no significant bilateral difference in ROI area was observed, minor geometric variation cannot be completely eliminated because of natural anatomical asymmetry.

For this reason, an additional methodological validation was performed using standardized 20 × 20 pixel square ROIs. Importantly, the square ROI analysis demonstrated the same direction of the right–left difference, with significantly higher FD values on the left side than on the right. This finding suggests that the principal observation was not solely attributable to differences in polygonal ROI geometry and supports the methodological consistency of the primary bilateral comparison. Although this complementary analysis cannot establish the biological origin of the observed difference, it reduces the likelihood that the principal finding reflects only ROI geometry.

Nevertheless, absolute FD values were consistently higher for square ROIs than for anatomically defined freehand ROIs on both sides. One possible explanation is that fixed square ROIs inevitably included varying proportions of adjacent cortical structures, dental roots, the nasal floor, and the anterior wall of the maxillary sinus, all of which may contribute to increased FD values.

Inter-observer reliability was excellent for both ROI approaches. Interestingly, agreement was slightly higher for anatomically defined freehand ROIs than for standardized square ROIs. Although fixed geometric ROIs are generally considered to improve standardization, both observers systematically excluded non-trabecular structures during freehand ROI selection and delineated the largest possible trabecular region according to predefined anatomical criteria. In contrast, the fixed dimensions of square ROIs limited the ability to avoid adjacent anatomical structures completely, and small differences in ROI positioning may therefore have resulted in greater observer-dependent variability despite the use of a standardized geometric template.

Therefore, these findings should be interpreted with consideration of the methodological sensitivity of FD analysis to ROI selection. Another limitation is that the observers were not blinded to image side during ROI selection and measurement. Because the anatomical side is inherently identifiable on panoramic radiographs, true side blinding was not feasible. However, predefined anatomical criteria, a standardized image-processing workflow, independent measurements performed by two observers, and reliability assessment using the ICC and Dahlberg formula were implemented to minimize potential observer bias. This methodological consideration should be taken into account when comparing the present results with studies using square, rectangular, or volumetric ROIs [11, 17, 20].

This study has several limitations. First, because the analyses were performed using panoramic radiographs, the two-dimensional nature of this imaging modality may limit complete representation of the three-dimensional trabecular architecture. Nevertheless, panoramic radiographs are routinely obtained in orthodontic practice, involve lower radiation exposure, and allow standardized bilateral assessment in relatively large samples. Because fractal analysis primarily evaluates trabecular organization rather than volumetric bone density, panoramic images are suitable for detecting relative side-related differences when ROI size and image parameters are adequately controlled. However, FD values may vary according to imaging modality, preprocessing workflow, thresholding strategy, and binarization methodology, indicating that fractal analysis remains partially workflow dependent [17, 20]. In addition, panoramic radiographs are susceptible to projection geometry, magnification variability, and anatomical superimposition. Accordingly, the present findings should be interpreted within the limitations of two-dimensional panoramic imaging and the standardized preprocessing protocol used in this study rather than as a direct representation of true trabecular architecture.

Importantly, the present study evaluated clinically healthy individuals, for whom CBCT in the absence of a diagnostic indication would not have been ethically justified. This approach is consistent with the ALARA (As Low As Reasonably Achievable) principle, which emphasizes minimizing exposure to ionizing radiation. Because the primary aim was to assess potential right–left variation in trabecular organization rather than three-dimensional morphology, panoramic radiographs were considered an appropriate and methodologically acceptable imaging modality.

In addition, because the primary objective was to evaluate subtle trabecular microstructural differences within the maxillary lateral incisor–canine region, only panoramic radiographs providing sufficiently clear bilateral visualization of this area were included. Radiographs with marked crowding, overlapping roots, unclear trabecular visualization, or inadequate delineation of the lateral incisor–canine interradicular region were excluded to allow selection of the largest representative ROI while minimizing superimposition-related distortion. Although this approach reduced the available sample size, it was considered necessary to improve anatomical consistency and the methodological reliability of the FD measurements.

Second, the sample consisted exclusively of clinically healthy individuals with Class I malocclusion and was predominantly composed of young adults, which may limit the generalizability of the findings to populations with different malocclusion patterns, skeletal relationships, age distributions, or dental and orofacial anomalies. Although the sample size was adequate for the primary paired comparison according to the power analysis, it may be insufficient for broader subgroup analyses. Therefore, the results should be interpreted as adequately powered for the primary bilateral comparison rather than for extensive stratified analyses. An additional limitation is that potential functional determinants of trabecular adaptation, including chewing-side preference, occlusal loading asymmetry, muscular dominance, and handedness, were not evaluated. Because trabecular bone responds to mechanical loading, these factors may also have influenced the observed side-related variation and should be considered in future studies.

Third, although the use of polygon-shaped freehand ROIs to minimize anatomical superimposition limited the selection of geometrically identical areas between sides, the absence of significant right–left differences in ROI area and grayscale parameters supports the methodological acceptability of this approach. Finally, because of the cross-sectional study design, developmental or temporal changes in trabecular organization could not be evaluated. Accordingly, these findings should be interpreted as a cross-sectional description of side-related variation rather than as evidence of its developmental origin or longitudinal clinical implications and should not be generalized beyond similarly selected clinically healthy populations.

Despite the statistically significant right–left difference, several methodological considerations should be taken into account when interpreting the findings. The observed effect size was small, indicating a modest magnitude of difference. Moreover, because FD measurements are sensitive to ROI delineation, image-processing workflows, and thresholding strategies, residual methodological variability cannot be completely excluded despite the use of a standardized protocol. Furthermore, because only the maxillary lateral incisor–canine region was evaluated, the findings should be regarded as region-specific observations rather than evidence of generalized maxillary asymmetry. Accordingly, the results should be interpreted primarily as an exploratory radiographic observation whose biological significance remains uncertain.

Within these limitations, this study provides several meaningful contributions to the literature. It was conducted in a relatively homogeneous sample, used a bilateral within-subject design, and applied a standardized image-processing workflow. Most importantly, it provides quantitative evidence of a subtle side-related difference in trabecular organization within the maxillary lateral incisor–canine region of clinically healthy adults. Future longitudinal studies incorporating ethically justified three-dimensional imaging and populations with specific dentoalveolar anomalies may help determine whether this finding has developmental, biomechanical, or treatment-related significance.

## Conclusion

Within the limitations of this retrospective radiographic study, a small but statistically significant right–left difference in fractal dimension values was detected within the maxillary lateral incisor–canine region. Although the observed effect size was small and its biological significance remains uncertain, the direction of the side-related difference was preserved using both anatomically defined freehand ROIs and standardized square ROIs, supporting the methodological consistency of the findings. In addition, excellent inter-observer reproducibility demonstrated the reliability of the proposed fractal analysis protocol. Accordingly, the present findings should be interpreted as a subtle radiographic difference detected under standardized methodological conditions rather than as definitive evidence of physiological maxillary asymmetry. Further studies incorporating multiple anatomical regions, functional variables, longitudinal follow-up, alternative image-processing strategies, and ethically justified three-dimensional imaging are required to clarify the biological and clinical relevance of side-related variation in maxillary trabecular organization.

## Data Availability

The datasets generated and/or analysed during the current study are not publicly available due to participant privacy and institutional data protection restrictions but are available from the corresponding author on reasonable request and with permission of Ankara University.
